# Elevated P75^NTR ^expression causes death of engrailed-deficient midbrain dopaminergic neurons by Erk1/2 suppression

**DOI:** 10.1186/1749-8104-4-11

**Published:** 2009-03-16

**Authors:** Kambiz N Alavian, Paola Sgadò, Lavinia Alberi, Srinivasa Subramaniam, Horst H Simon

**Affiliations:** 1 Interdisciplinary Centre for Neuroscience, Department of Neuroanatomy, Ruprecht-Karls-Universität, 69120 Heidelberg, Germany; 2 Harvard Medical School, Neuroregeneration Labs, MRC 1, McLean Hospital, Mill St, Belmont, MA 02478, USA; 3 Paola Sgadò, Neurogenetics Laboratory, Child Neurology Unit, Pediatric Hospital A Meyer, Piazza di Careggi, 50139 Florence, Italy; 4 The Johns Hopkins Institute for Cell Engineering, Department of Neurology, North Broadway Street, BRB 720, Baltimore, MD 2120, USA; 5 Department of Neuroscience, Johns Hopkins Medical School, N Wolfe Street, Baltimore, MD 21210, USA

## Abstract

**Background:**

The homeodomain transcription factors *Engrailed-1 *and *Engrailed-2 *are required for the survival of mesencephalic dopaminergic (mesDA) neurons in a cell-autonomous and gene-dose-dependent manner. Homozygote mutant mice, deficient of both genes (*En1-/-;En2-/-*), die at birth and exhibit a loss of all mesDA neurons by mid-gestation. In heterozygote animals (*En1+/-;En2-/-*), which are viable and fertile, postnatal maintenance of the nigrostriatal dopaminergic system is afflicted, leading to a progressive degeneration specific to this subpopulation and Parkinson's disease-like molecular and behavioral deficits.

**Results:**

In this work, we show that the dose of *Engrailed *is inversely correlated to the expression level of the pan-neurotrophin receptor gene *P75*^*NTR *^(*Ngfr*). Loss of mesDA neurons in the *Engrailed*-null mutant embryos is caused by elevated expression of this neurotrophin receptor: Unusually, in this case, the cell death signal of P75^NTR ^is mediated by suppression of Erk1/2 (extracellular-signal-regulated kinase 1/2) activity. The reduction in expression of *Engrailed*, possibly related to the higher levels of P75^NTR^, also decreases mitochondrial stability. In particular, the dose of *Engrailed *determines the sensitivity to cell death induced by the classic Parkinson-model toxin MPTP and to inhibition of the anti-apoptotic members of the Bcl-2 family of proteins.

**Conclusion:**

Our study links the survival function of the *Engrailed *genes in developing mesDA neurons to the regulation of *P75*^*NTR *^and the sensitivity of these neurons to mitochondrial insult. The similarities to the disease etiology in combination with the nigral phenotype of *En1+/-;En2-/- *mice suggests that haplotype variations in the *Engrailed *genes and/or *P75*^*NTR *^that alter their expression levels could, in part, determine susceptibility to Parkinson's disease.

## Background

Mesencephalic dopaminergic (mesDA) neurons are the main source of dopamine in the mammalian central nervous system. They are located in three distinct nuclei, substantia nigra pars compacta, ventral tegmentum and retrorubral field. Their main innervation targets are the basal ganglia, where they play key roles in the control of emotion, motivation and motor behavior, documented by their connection to schizophrenia, addiction and, most prominently, to Parkinson's disease (PD) [1,2]. The main characteristic of PD is the slow progressive loss of dopaminergic neurons in the substantia nigra pars compacta, causing diminished release of dopamine in the caudate putamen and debilitating motor deficits. Although the molecular causes for the selective vulnerability of this neuronal population are hardly understood, multiple lines of evidence suggest mitochondrial dysfunction as a major contributing factor [3] and apoptosis as the executioner of cell death [4]. Several mutations associated with familial forms of PD encode mitochondrial proteins [5] and neurotoxins specific to the nigrostriatal system – MPTP (1-methyl-4-phenyl-,1,3,6-tetrahydropyridine), 6-hydroxydopamine and rotenone – cause mitochondrial damage as inhibitors of complex-I of the electron transport chain [6]. Mitochondrial insult can cause oxidative stress by production of reactive oxygen species, leading to increased permeability of the mitochondrial membrane, release of pro-apoptotic molecules, including cytochrome-C, into the cytoplasm and, subsequently, to activation of caspases and induction of apoptosis [7].

Neuronal cell death can be a result of neurotrophin deficiency. Action of the neurotrophins, consisting of nerve growth factor (NGF), brain-derived neurotrophic factor (BDNF), neurotrophin (NT)4/5 and NT3, is mediated via a set of specific tyrosine kinase (Trk) receptors (TrkA, B, C) and a common receptor, P75^NTR ^(Ngfr). While the Trk receptors signal survival, P75^NTR ^can relay a survival or cell death signal, depending on the cellular context and the molecular form of the ligand [8]. The pro-survival function of neurotrophins and their receptors has been mainly attributed to the downstream effect of phosphotidyl inositol-3 kinase (PI3K) and the extracellular-signal-regulated kinase 1/2 (Erk1/2) pathways [9], whereas cell death signaling via P75^NTR ^is mediated by phosphorylation of c-Jun N-terminal kinase (JNK) and BH3-only members of the Bcl-2 family [10].

The homeodomain transcription factors *Engrailed-1 *(*En1*) and *Engrailed-2 *(*En2*) are required for the survival and maintenance of mesDA neurons in a cell-autonomous and gene-dose-dependent manner, demonstrated by *in vitro *cell mixing experiments, RNA interference (RNAi) and analysis of chimeric mice [11-13]. In *Engrailed *double mutant mice (*En1-/-;En2-/-*; from here on *En*^*DM*^), the mesDA neurons are generated and begin to express their neurotransmitter phenotype but then die by apoptosis between embryonic day (E)12 and E14, the stage when *Engrailed *expression starts in their wild-type counterparts [11,12]. The intermediate genotypes between *En*^*DM *^and wild type show various degrees of cell loss in the mesDA system. Most interestingly, mice heterozygous null for *En1 *and homozygous null for *En2 *(*En1+/-;En2-/-*; from here on *En*^*HT*^), which are viable and fertile, exhibit a slow progressive loss of nigral dopaminergic neurons within the first two months after birth, resulting in diminished storage and release of dopamine in the striatum and in PD-like motor deficiencies [14].

We show here that *Engrailed*-deficiency in mesDA neurons leads to elevated *P75*^*NTR *^expression that is causal for cell death if the neurons are null for *En1 *and *En2*. The death signal is mediated by the suppression of Erk1/2 activity. Probably linked to the P75^NTR ^elevation, the dose of the *Engrailed *genes also determines the sensitivity to mitochondrial insult.

## Materials and methods

### Animals

The generation of the *En2 *null mutant and the *En1tau-LacZ *'knock-in' mice have been previously described [15,16]. The *En2 *mutants with an original mixed genetic background of 129 and Swiss Webster were crossed three times into a C57/BL6 background. The line was bred as *En1+/tlZ;En2-/- *at the central animal facility, University of Heidelberg.

### Quantitative RT-PCR

Quantitative RT-PCR reactions were performed according to the manufacture in a 7000 Sequence Detection System from Applied Biosystems (Foster City, CA, USA) using pre-formulated 'assays on-demand' and calculating the results with the comparative cycle time (CT) method. The pre-formulated 'assays on demand' had the following identification tag: Mm00446294_m1 for Ngfr (P75^NTR^); as standard control Mm00507222_s1 ribosomal protein S18; Mm00435617_m1 for phosphoglycerate kinase 1 (Pgk1); and Mm00446973_m1 for TATA box binding protein (Tbp). The dissected ventral midbrains were homogenized, the RNA isolated and reverse transcribed using random hexamers to initiate transcription. Each of the individual PCR reactions was done in triplicate and at least two of three standard controls were run in parallel. Each of the experimental sets consisted of RNA from different pools of mutant and littermate control as well as RNA from Tet-On induced *Engrailed *expressing N2A cells and control.

### Cell culture

All primary cell cultures were performed using E12.5 mouse embryos. *En*^*DM *^embryos were distinguished from the other genotypes in the litter by their midbrain/hindbrain morphology [11], which was occasionally verified by PCR. Distinction between *En2-/- *and *En1+/tlZ;En2-/- *embryos was achieved by incubation of the limb buds in X-Gal solution at 37°C (40 mg/ml X-Gal (Sigma-Aldrich; Munich, Germany) in 5 mM K_3_Fe(CN), 5 mM K_4_Fe(CN)_6_x3H_2_O, 1 mM MgCl_2 _in phosphate-buffered saline). For the cell culture, the neural tubes were dissected and ventral midbrains were isolated. The tissue was then dissociated using trypsine (Invitrogen; Karlsruhe, Germany). The preparation of laminin (Sigma) coated coverslips was described elsewhere [11]. The medium was DMEM-F12 supplemented with 5% fetal calf serum, 0.25% bovine serum albumin (Sigma), 33 mM glucose, 50 U/ml penicillin, 50 U/ml streptomycin, and 1% Fungizone (Invitrogen). The cells were seeded at approximately 150,000 per cover slip and incubated at 37°C. After 36 hours, *En*^*DM *^mutant cells were fixed in 4% paraformaldehyde and processed for immunostaining. The typical numbers of tyrosine hydroxylase (TH)-positive cells per cover slip were between 100 and 300 cells if wild-type or heterozygote mutant tissue was dissociated. The numbers of TH-positive neurons was always significantly lower if the dissociated ventral midbrains were derived from E12 *En*^*DM *^embryos. To obtain comparable numbers for mutant and wild-type experiments, all cell counts were normalized against each of the controls. Numbers presented in the results section (n) refer to the number of experiments. Each of the experimental conditions is represented by at least three cover slips in each experiment. The optimum concentration for the toxic substances was determined by titration and checking for an intermediate rate of survival (between 30% and 60%) in cultures of mixed genotypes (*En2-/- *and *En1+/-;En2-/-*). For induction of cell death, serum was withdrawn from the medium after 48 hours and the cultures were treated with the compounds HA14-1, chelerythrine chloride (Axxora, San Diego, CA)), prima-1, Apoptosis Activator-2, tumor necrosis factor (TNF)α and 1-methyl-4-phenylpyridinium (MPP^+^). Used concentrations, solvents, durations of treatment and vendor sources are provided in Additional file 1[17-33]. The number 'n' corresponds to the number of individual experiments conducted.

### Design and transfection of siRNA oligos

The design of the 21-mer RNAi oligonucleotides was carried out at Biomers.net (Ulm, Germany) and in accordance with the protocol by Ebashir *et al*. [34]. Both RNA duplexes targeted the coding sequence of *P75*^*NTR *^(A, sense ACAGAACACAGUGUGUGAA(dTdT) and anti-sense UUCACACACUGUGUUCUGU(dTdT); B, sense CAUUCCGACCGCUGAUGUUCU(dTdT) and anti-sense AACAUCAGCGGUCGGAAUGUG(dTdT)). For coupling with Penetratin-One (QBiogene; Strasbourg, France), the sense strand was modified with a thiol group on the 5' end. Tris-2-carboxyethylphosphine (TCEP; 1 μl; Pierce; Bonn, Germany) was added to 224 μl of the small interfering RNA (siRNA; stock solution, 900 μM) and incubated for 15 minutes at room temperature. Then, 25 μl of Penetratin-1 was added, mixed and incubated for 5 minutes at 65°C following by 1 hour at 37°C. Penetratin-1 solution was reconstituted to 2 mg/ml (≈0.8 mM) in sterile water. A stock solution of 20 mM TCEP in sterile RNAse/DNAse free water was made. The aliquots were frozen at -80°C. The Penetratin-coupled siRNA oligos were heated to 65°C for 15 minutes and 3 μl of the mix was dissolved in complete growth medium. As controls, we used Penetratin-1 coupled double-stranded RNA oligos directed against Maged1 (Nrage) (UAACUUGAAUGUGGAAGAG(dTdT) and CUCUUCCACAUUCAAGUUA(dTdT)) and the randomly generated Scramble I Duplex (Dpharmacon; Heidelberg, Germany) sense CAGTCGCGTTTGCGACTGG and antisense CCAGTCGCAAACGCGACTG. Both had no effect on the survival rate of control or *En*^*DM *^mesDA neurons. Alternatively, uncoupled double-stranded RNA oligos with the same sequences were transfected using HiPerfect (Qiagen; Hilden, Germany) in accordance with the manufacturer's protocol.

### Immunohistochemistry and western blot analysis

The immunohistochemistry and western blot analysis was done according to the protocol described elsewhere [35]. All the phospho-specific antibodies were purchased from Cell Signaling Technology, the neurotrophin and TH antibodies from Chemicon (Molsheim, France) and the antibodies for neurotrophin receptors from Santa Cruz Biotechnology (Heidelberg, Germany).

### Statistical analysis

Values are expressed as mean ± standard error. Differences between means were analyzed by using a paired, two-tailed Student *t*-test. All shown *p*-values are rounded up at the third or fourth digit.

## Results

### Elevated P75^NTR ^expression in absence of Engrailed genes

**Figure 1:**
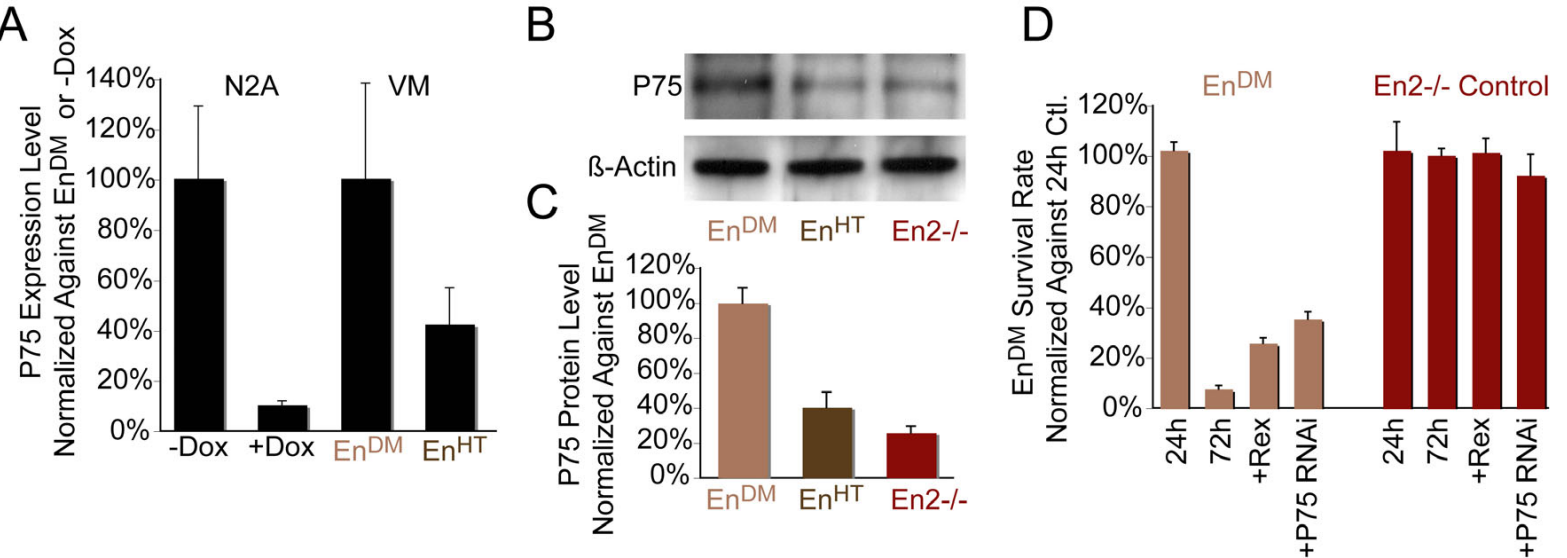
**Elevated *P75*^*NTR *^expression is causal for cell death**. **(A) **Quantitative RT-PCR of ventral midbrain tissue (VM) derived from *En*^*DM *^(*En1-/-;En2-/-*) and *En*^*HT *^E12 embryos, and of *En1*-expressing N2A cells inducible by doxycycline (Dox). *P75*^*NTR *^expression is inversely correlated with *En1 *expression levels in tissues and cell lines. **(B, C) **Western blot analysis of the ventral midbrain tissue shows the same relationship between P75^NTR ^protein levels and *En1 *expression. Each active *En1 *allele decreases the P75^NTR ^expression level (n = 3, *p *= 0.002). **(D) **Ventral midbrain cultures derived from *En*^*DM *^and *En2-/- *embryos. Silencing of *P75*^*NTR *^by double-stranded RNA oligos and application of P75^NTR^-inhibiting antibody (Rex) increases the survival rate of *En*^*DM *^mesDA neurons compared to untreated control (Ctl) or after treatment with scrambled RNA oligos (n ≥ 6, *p *< 0.01). Error bars indicate standard error.

A genome-wide expression analysis, using microarrays on *En1 *inducible N2A cell line, identified the NGF receptor *P75*^*NTR *^as downstream of the *Engrailed *transcription factors (data not shown). To confirm the microarray, we examined by quantitative RT-PCR the levels of *P75*^*NTR *^in the cell line as well as in ventral midbrain tissue in relationship to *En1 *expression. As a result of *En1 *induction in N2A cells, the endogenous expression of *P75*^*NTR*^ decreased by 10-fold (9.9 ± 1.9%, *p *< 0.001, n = 8). Likewise, the ventral midbrain tissue, derived from control littermates (*En2-/-*) expressed almost 2.5-fold less *P75*^*NTR *^(42% ± 14.0%, *p *< 0.001, n = 6) than *En*^*DM *^mutants (Figure 1A). The western blot analysis confirmed the latter results; ventral midbrain from *En2-/- *and *En*^*HT *^embryos contained 74.4 ± 4.4% (*p *< 0.001, n = 3) and 60.2 ± 8.8% (*p *= 0.002, n = 3) less P75^NTR ^protein, respectively, than the same tissue from *En*^*DM *^littermates (Figure 1B,C).

Since P75^NTR ^can mediate cell death in neurons [8], we began to investigate whether its elevated expression is causal for the death of mesDA neurons in *En*^*DM *^embryos. In order to functionally interfere with P75^NTR^, we applied an activity-blocking antibody (Rex) [28] to primary ventral midbrain cell cultures. This antibody increased the survival rate from 7.5 ± 1.24% to 34.8 ± 4.6% (*p *< 0.001, n = 6; Figure 1D). Furthermore, to lower *P75*^*NTR *^expression levels in the mutant neurons, we applied specific Penetratin-coupled siRNA duplexes [36]; 72 hours after transfection, the total P75^NTR ^protein was reduced by 83.2 ± 6.3% (*p *= 0.05, n = 3; western blot not shown) and the survival rate increased from 7.5 ± 1.24% to 25.1 ± 2.1% (*p *< 0.001 n = 16) (Figure 1D). These data suggested that elevated expression of *P75*^*NTR *^is the direct cause of the induction of apoptosis in *Engrailed*-deficient mesDA neurons.

**Figure 2:**
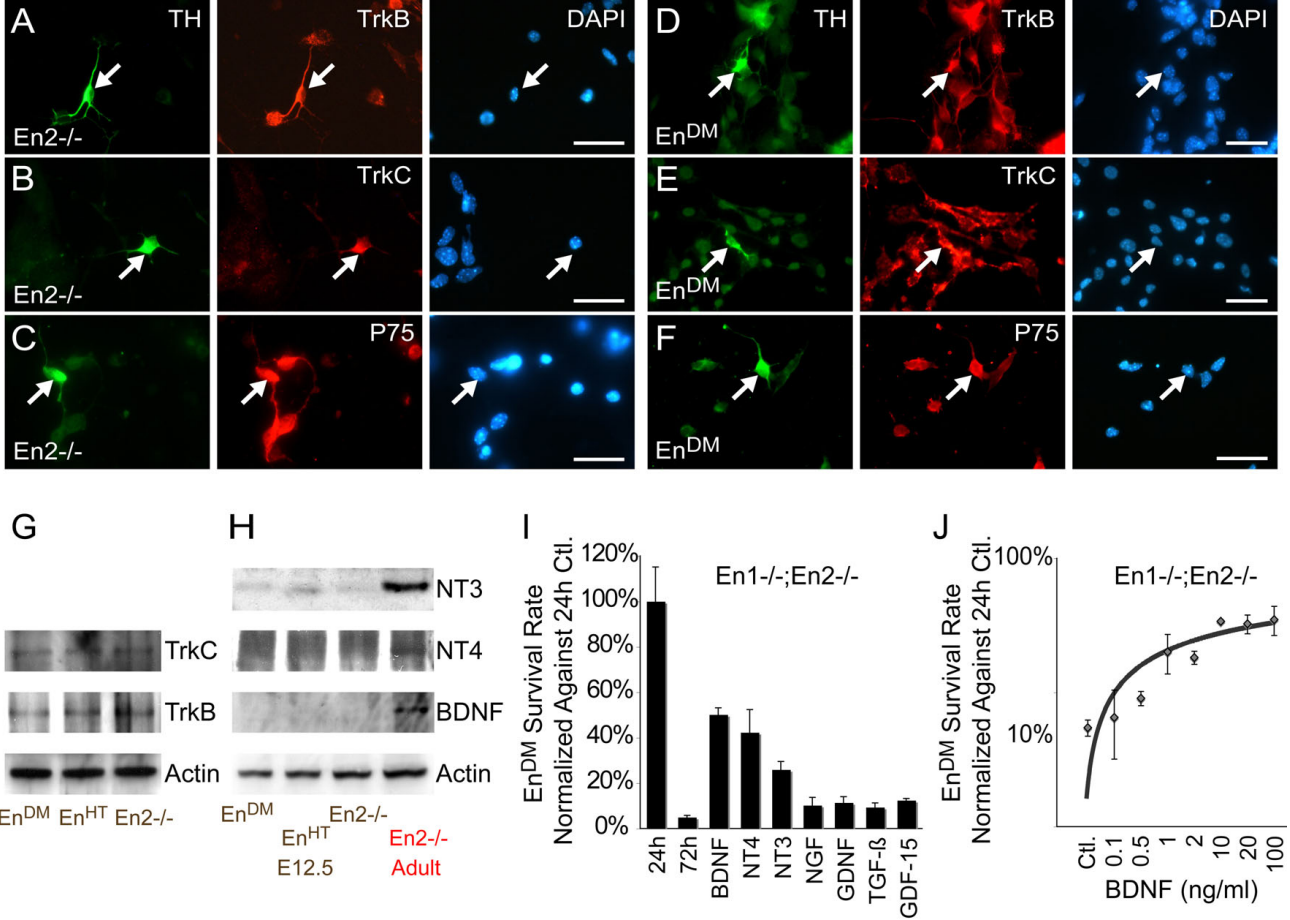
**Loss of *Engrailed *induces neurotrophin requirement in mesDA neurons**. **(A-F) **Double immunohistochemistry on dissociated cells derived from *En2-/- *(A-C) and *En1-/-;En2-/- *(*En*^*DM*^) (D-F) E12 ventral midbrain using antibodies against tyrosine kinase (Trk)B (A, D), TrkC (B, E), P75^NTR^(C, F) and TH (green) counterstained with DAPI. TrkB, TrkC and P75^NTR^ are expressed by TH+ cells from both genotypes; however, the immunohistochemistry is not sensitive enough to detect differences in P75^NTR ^expression between genotypes. **(G, H) **Western blot of ventral midbrain tissue derived from different *Engrailed *genotypes. The two Trk receptors do not depend on *Engrailed *expression (G). Brain-derived neurotrophic factor (BDNF), neurotrophin (NT)4 and NT3 are not expressed in E12 ventral midbrain tissue, but they are in the adult (H). **(I) **Treatments (>10 ng/ml) for 72 hours with TrkB/C-specific neurotrophins – BDNF, NT4 and NT3 – greatly increases the survival rate of *En*^*DM *^mesDA neurons (n ≥ 6; *p *< 0.001), whereas nerve growth factor (NGF), glial cell line-derived neurotrophic factor (GDNF), transforming growth factor (TGF)-β and growth differentiation factor (GDF)-15 do not significantly alter survival rate. **(J) **Dose response curve: BDNF concentration plotted against survival rate showing saturation at approximately the 10 ng/ml. Scale bars: 25 μm. Error bars indicate standard error. Ctl, control.

P75^NTR ^mediates dual, opposing functions of cell survival and death, controlled by the presence or absence of neurotrophins. For the anti-apoptotic function, neurotrophins require their cognate Trk receptors as heterodimerization partners for P75^NTR ^[8]. In order to assess a potential role of the Trk/P75^NTR ^system during the course of cell loss, we determined the expression of the Trk-receptors in E12 mesDA neurons. TrkC and TrkB, but not TrkA, were detectable by immunohistochemistry and western blot at equal levels in wild type and *En*^*DM *^mutants (Figure 2A–G).

**Figure 3:**
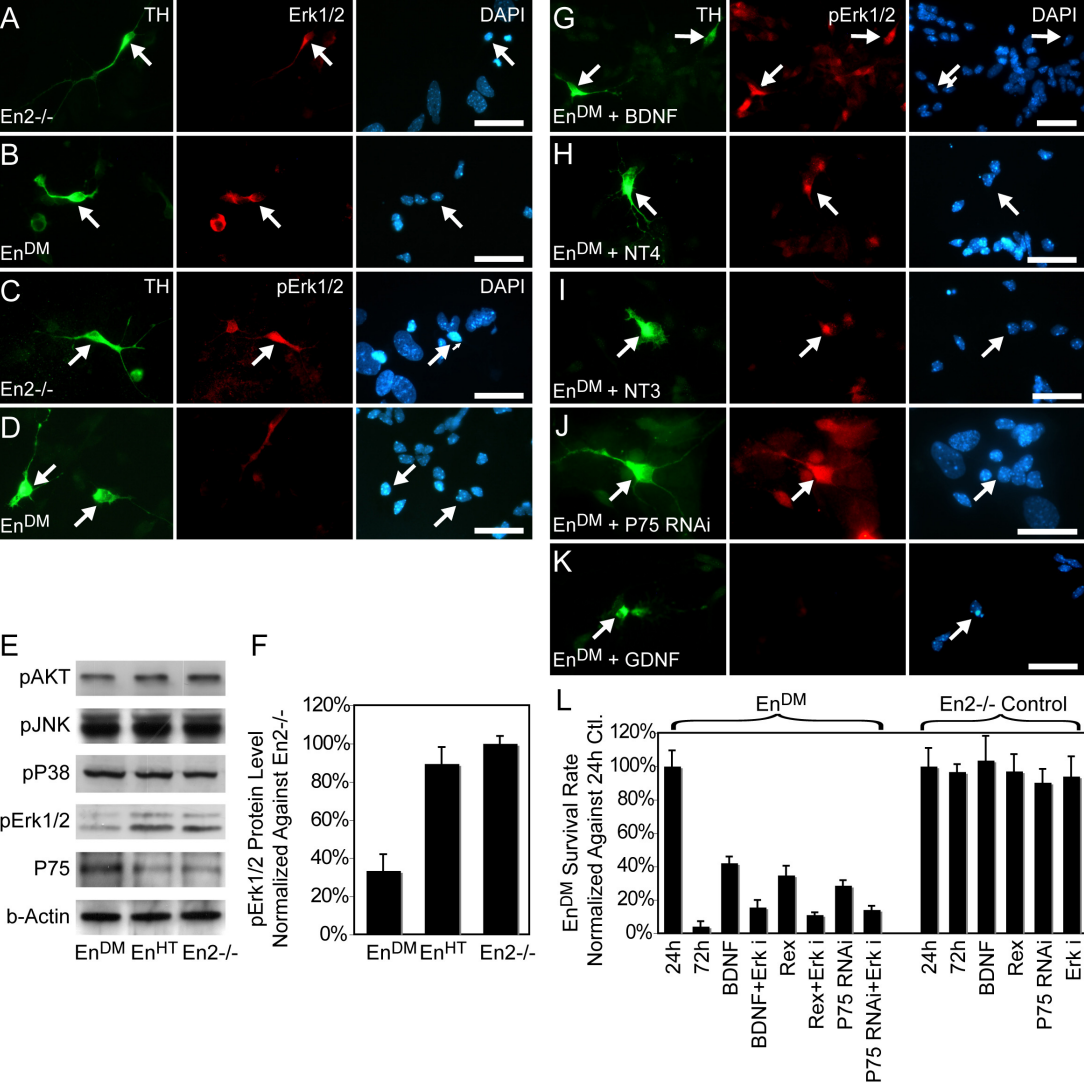
**Differential activation of Erk1/2 in mesDA neurons**. **(A-D, G-K) **Immunohistochemistry of E12 ventral midbrain cell culture stained against TH (green), total Erk1/2 protein (red) (A, B) and phosphorylated Erk1/2 (red) (C-D, G-K). (A-D) While Erk1/2 protein is present in mesDA neurons of both genotypes (A, B), it is only phosphorylated in *En2-/- *mesDA neurons (C) and not in the *En1-/-;En2-/- *(*En*^*DM*^) counterparts (D). **(E-I) **Erk1/2 becomes activated in *En*^*DM *^mesDA neurons after treatment with the survival-inducing neurotrophins, brain-derived neurotrophic factor (BDNF), neurotrophin (NT)4 and NT3, or after silencing of P75^NTR ^(RNA interference (RNAi)) (G-J), but not when glial cell line-derived neurotrophic factor (GDNF) is applied (I). (E) Western blot of E12 ventral midbrain tissue confirms the immunohistochemical finding of differential phosphorylation between genotypes and shows that neither AKT, part of the phosphotidyl inositol-3 kinase pathway, nor other mitogen-activated protein kinases, such as JNK and P38, are differentially activated. (F) Quantification of phosphorylated Erk1/2 in western blot normalized against *En2-/- *tissue. **(L) **Number of TH-positive cells in *En*^*DM *^and *En2-/- *ventral midbrain cultures after 72 hours, treated with the 400 nM Mek inhibitor U0126 in conjunction with BDNF, Penetratin-coupled *P75*^*NTR *^double-stranded RNA oligonucleotides and the P75^NTR ^inhibiting antibody (Rex). Numbers are normalized against untreated cultures at 24 hours. The rescue effect is significantly reduced when the *En*^*DM *^cultures are treated with the Erk1/2 inhibitor. Scale bars: 25 μm. Error bars indicate standard error. Ctl, control.

The up-regulation of *P75*^*NTR *^and the presence of Trk receptors suggested that *Engrailed *deficiency introduces a neurotrophin requirement to the E12 mesDA neurons that cannot be satisfied at this age, since the neurotrophins specific to TrkB and TrkC – that is, BDNF, NT4 and NT3 – are not expressed in the E12 ventral midbrain as they are in the adult (Figure 2H). To test this hypothesis, we applied saturating concentrations of BDNF, NT4 and NT3 to ventral midbrain cultures. After 72 hours, 50.2 ± 2.9% (*p *< 0.0001, n = 27), 42.3 ± 10.1% (*p *< 0.001, n = 9) and 26.0 ± 3.5% (*p *< 0.001, n = 9), respectively, of the otherwise dying *En*^*DM *^mesDA neurons were still present in the cultures (Figure 2I). The addition of BDNF to the control littermate cultures demonstrated that this was due to an elevated survival rate and not attributable to a higher rate of precursor cell proliferation (Figure 3L). As expected from the lack of TrkA, application of its ligand, NGF, did not change the survival rate significantly. To test the specificity of BNDF, NT3 and NT4, we applied glial cell line-derived neurotrophic factor (GDNF), growth differentiation factor (GDF)-15 and transforming growth factor (TGF)-β to the mutant cultures, all known survival factors for mesDA neurons [37-39]. Similar to NGF, none of them prevented the death of the *Engrailed*-deficient mesDA neurons (Figure 2I). Furthermore, the linear dose-response trend-line of the survival effect of BDNF (Figure 2J) indicated a Kd value of approximately 2.5 nM, which corresponds with the reported affinity of BDNF for the TrkB/P75^NTR^complex [40].

### The survival-mediating role of Erk1/2

The survival function of neurotrophins has been mainly attributed to Erk1/2 and PI3K pathways [41]. As the next step, we examined whether these pathways play a role in arbitrating the effect of the neurotrophins on *Engrailed*-deficient mesDA neurons and whether they intersect with the molecular events regulated by *Engrailed *expression. Therefore, we studied activation of the two pathways, using antibodies against the phosphorylated forms of Erk1/2 and AKT [41]. Immunohistochemistry showed that while total Erk1/2 protein was present in both *En2-/- *and *En*^*DM *^mesDA neurons, it was activated only in the wild-type-like cells and not in *En*^*DM *^mesDA neurons (Figure 3A–D). Western blot analysis of ventral midbrain tissue showed a similar effect; Erk1/2 activity in E12 *En*^*DM *^ventral midbrain was reduced by 66.8 ± 18.2% (*p *= 0.02, n = 3) in comparison to the same tissue derived from their *En2-/- *littermates (Figure 3E,F). In contrast to Erk1/2, we did not detect differential activation of AKT in the western blot (Figure 3E). Additionally, we could not detect signs of differential activation of other components of the mitogen-activated protein kinase (MAPK) pathways, such as JNK or P38 (Figure 3E).

**Figure 4:**
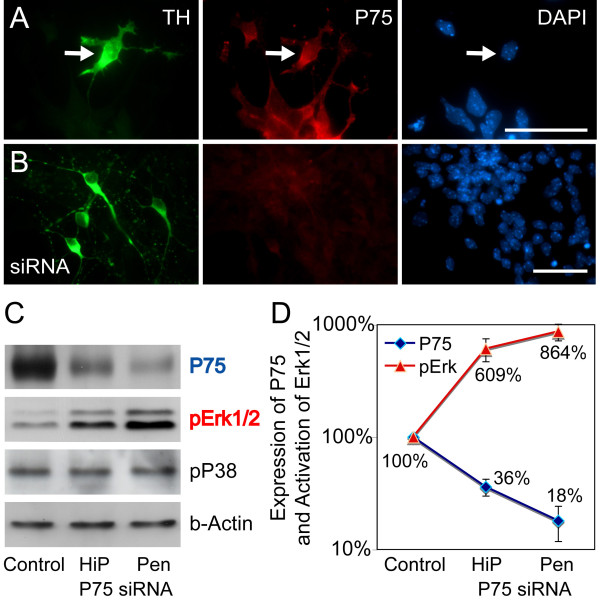
**Erk1/2 activation is inversely correlated with P75^NTR ^expression**. **(A, B) **Immunohistochemistry on E12 dissociated ventral midbrain cultures derived from *En*^*HT *^E12 embryos stained against TH (green) and P75^NTR^. P75^NTR ^expression is absent in cultures treated with Penetratin-coupled double-stranded RNA oligos. **(C) **Western blot of ventral midbrain cultures using two methods of RNA transfection with different efficiencies: HiPerfect (HiP) and Penetratin-coupled RNA oligos show increase in phosphorylated Erk1/2 after silencing of *P75*^*NTR*^, but no changes in phosphorylation of P38. **(D) **Quantification of P75^NTR ^expression and Erk1/2 phosphorylation after transfection with P75^NTR ^double-stranded RNA oligonucleotides, normalized against untreated *En*^*HT *^cultures, showing the inverse correlation between the two parameters. Scale bars: 25 μm. Error bars indicate standard error.

The loss of Erk1/2 phosphorylation suggested that this MAPK pathway was differentially activated in *En*^*DM *^mesDA neurons in response to a survival or death signal. To investigate this hypothesis, we examined the state of activation of Erk1/2 after application of the survival factors. The addition of BDNF, NT4 and NT3, and the knock-down of *P75*^*NTR *^by siRNA oligonucleotides all caused phosphorylation of Erk1/2 (Figure 3G–J); however, MAPK was not activated after application of NGF, GDNF, TGF-β or GDF-15 (for example, see Figure 3K). If the loss of Erk1/2 activity is the primary cause of cell death, when P75^NTR ^is elevated in mesDA neurons, and this correlation is not accidental, then inhibition of the Erk1/2 pathway should interfere with the rescue by the neurotrophins and by P75^NTR ^inhibition or silencing. To test this, we concurrently treated the cultures with U0126 [42], an inhibitor of the MAPK kinase upstream of Erk1/2, MEK1/2 [32], at a concentration (400 nM) not toxic to the wild-type (*En2-/-*) neurons. The inhibition of Erk1/2 by U0126 significantly reduced the rescue effect of all three survival factors; from 42.2 ± 3.1% to 11.7 ± 3.5% (*p *= 0.002) for BDNF, from 34.7 ± 4.7% to 8.3 ± 2.7% (*p *< 0.0001) for Rex and from 25.4 ± 2.7% to 10.8 ± 1.9% (*p *= 0.009) after *P75*^*NTR *^silencing (control mutant 4.2 ± 2.2%, *p *= 0.01, n = 4 for all experiments) (Figure 3L).

To elaborate further on the correlation between the expression of *P75*^*NTR *^and the state of phosphorylation of Erk1/2, we silenced the *P75*^*NTR *^expression in *En*^*HT *^ventral midbrain cultures by RNAi, using two methods with different transfection efficiencies (the lipophilic transfection reagent HiPerfect, and Penetratin-coupled oligos). The former reduced *P75*^*NTR *^expression levels, on average, by 63.3 ± 2.0% (*p *= 0.011, n = 3) and the latter by 82.2 ± 6.3% (*p *= 0.05, n = 3). This, in turn, caused 6.09 ± 1.40-fold (*p *= 0.01, n = 3) and 8.64 ± 1.45-fold (*p *= 0.008, n = 3) increases, respectively, in the phosphorylation of Erk1/2 (Figure 4A–D).

### Sensitivity to mitochondrial dysfunction and Engrailed expression level

**Figure 5:**
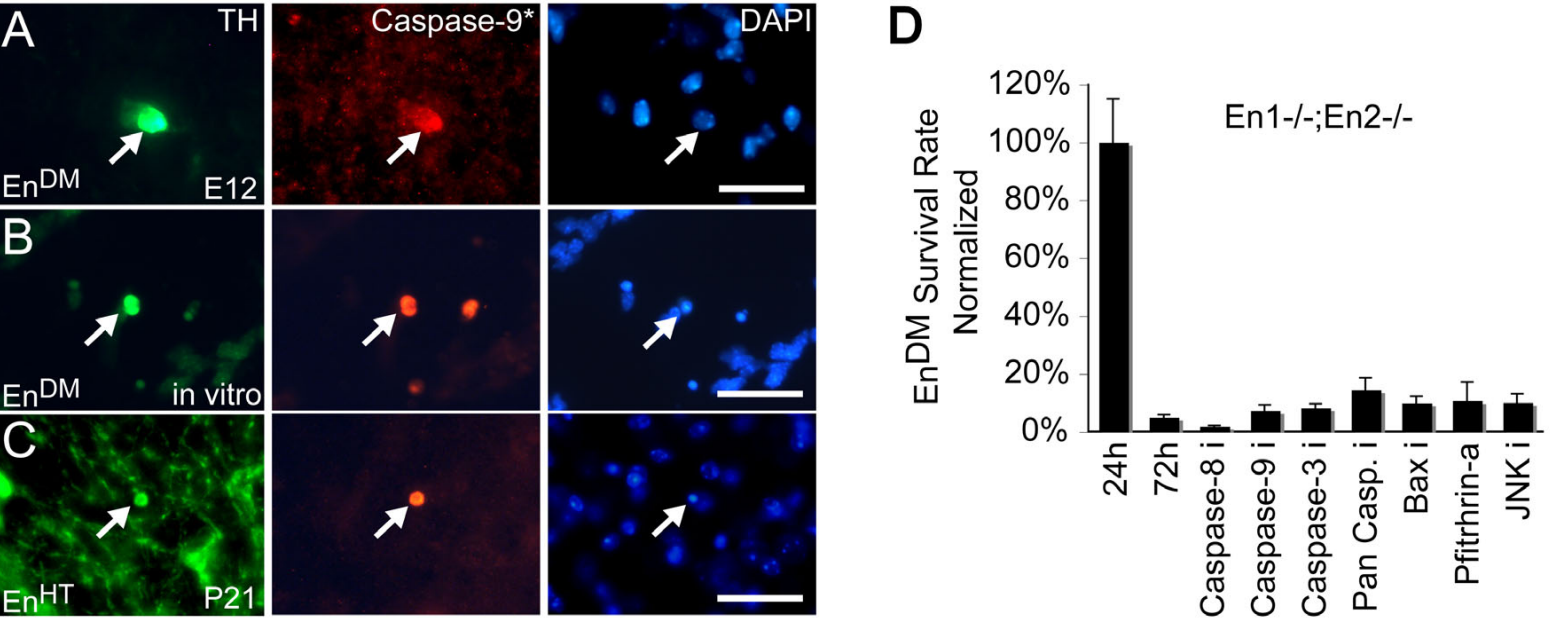
**Mitochondrial apoptosis in *Engrailed*-deficient mesDA neurons**. **(A-C) **TH and activated caspase-9 immunohistochemistry on ventral midbrain coronal sections of E13 *En1-/-;En2-/- *(*En*^*DM*^) embryos (A), of *En1+/-;En2-/- *(*En*^*HT*^) postnatal day 20 brain (C), and of *En*^*DM *^E12 cell culture (B) counterstained with the nuclear marker DAPI. Dying TH-positive neurons (arrows) exhibit small rounded cell bodies and signs of apoptosis, that is, activated caspase-9 and pyknotic nuclei (DAPI). **(D) ***En*^*DM *^ventral midbrain cultures treated for 72 hours with inhibitors for caspases-3, -8 and -9, a pan-caspase inhibitor, z-vad-fmk, the BAX inhibitor Ku70, the P53 inhibitor Pfithrin-α, and the JNK inhibitor SP600125. None of the treatments significantly changed the survival rate of *En*^*DM *^mesDA neurons. The number of surviving TH-positive cells was normalized in each case against untreated cultures 24 hours after dissociation. Scale bars: 25 μm. Error bars indicate standard error.

P75^NTR ^signaling often mediates cell death via induction of the mitochondrial (intrinsic) pathway of apoptosis [43,44]. To further assess whether the elevated level of *P75*^*NTR *^expression in the *Engrailed*-deficient mesDA neurons is causal for demise of the cells, we investigated the dying neurons for signs of this pathway. We had previously reported that loss of *Engrailed *expression in mesDA neurons causes activation of caspase-3 [11,14], an effector caspase, triggered by the intrinsic or extrinsic pathways of apoptosis [45,46]. The intrinsic death pathway involves release of cytochrome C from the mitochondria, which participates in formation of apoptosome, which is required for activation of caspase-9. We detected small, rounded TH-positive cells with active caspase-9, and pyknotic, fragmented nuclei in the ventral midbrain of *Engrailed*-deficient E13 embryos as well as in *En*^*HT *^mice during the postnatal stages of nigral cell loss (postnatal day 20; Figure 5A–C) confirming our hypothesis that the pathway downstream of P75^NTR ^signaling must be the cause of the demise of these neurons in the absence of the *Engrailed *genes [43].

**Figure 6:**
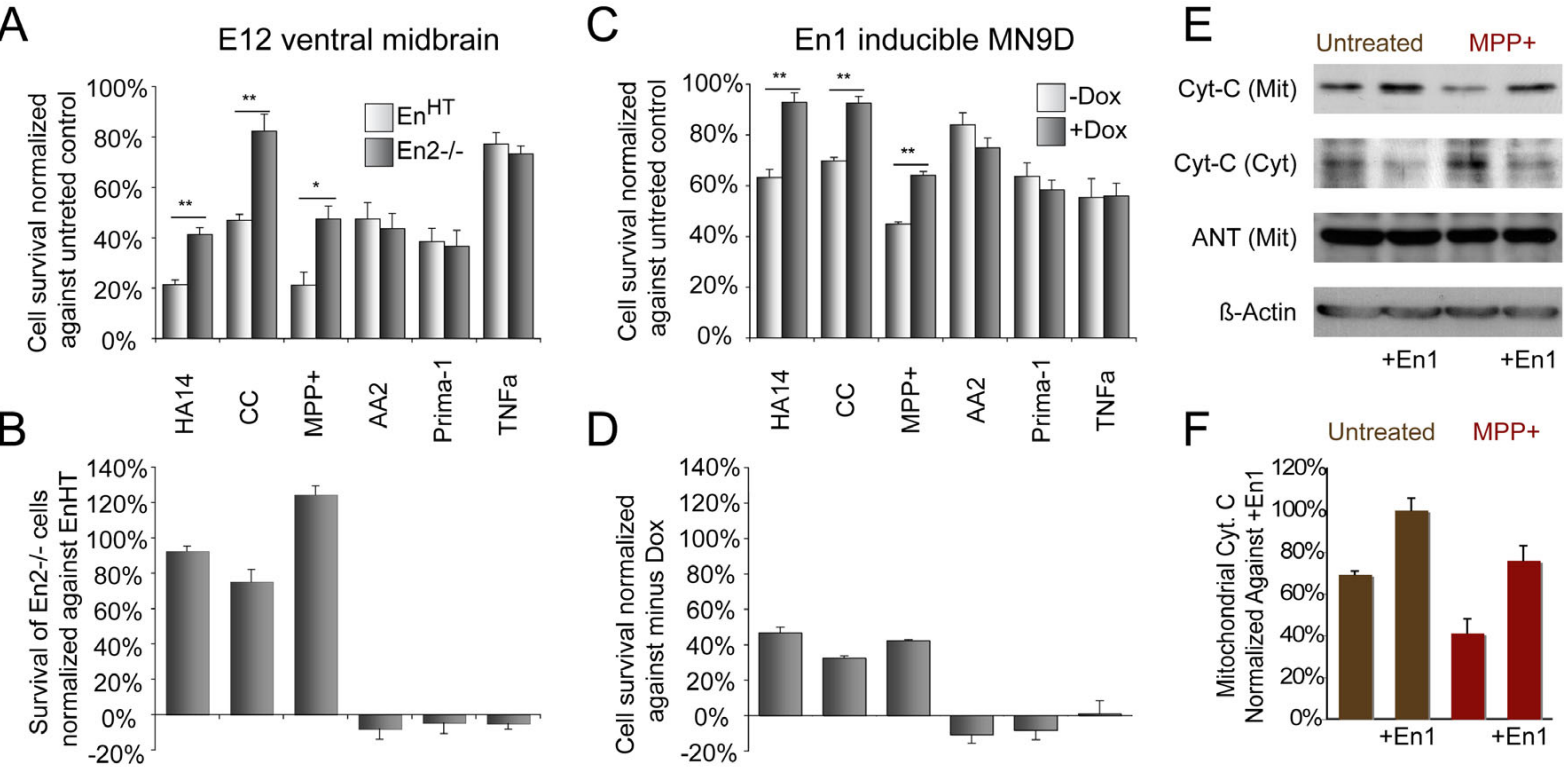
**The sensitivity to induction of the intrinsic pathway of apoptosis correlates with *En1 *expression**. **(A-D) **Ventral midbrain cultures 24 hours after application of apoptosis-inducing compounds. Charts of *En*^*HT *^(*En1+/-;En2-/-*) and *En2-/- *E12 cultures depicting the number of surviving TH-positive cells (A, B) and cultures of *En1*-inducible MN9D cells depicting cell survival measured by cell proliferation assay (C, D). Surviving cells were normalized against untreated *En*^*HT *^cultures (A) or untreated non-induced MN9D cells (C), treated *En*^*HT *^cultures (B) or treated non-induced MN9D cells (D). Higher *Engrailed *expression reduced the cell death rate after MPP^+^, HA14-1 and chelerythrine chloride (CC) treatment, whereas the rate of cell survival after application of the tumor necrosis factor alpha (TNFa), Prima-1 and Apoptosis Activator-2 (AA2) does not correlate with the level of *En1 *expression. Dox, doxycycline. **(E) **Western blot analysis of mitochondrial and cytoplasmic protein fractions of MN9D cells 72 hours after *En1 *induction and 24 hours after MPP^+ ^treatment. **(F) **Proportion of cytochrome C (Cyt-C) in cytosol is lower in *En1*-expressing MN9D cells before and after MPP^+ ^treatment. Scale bars: 25 μm. Error bars indicate standard error. 6 ≤ n ≤ 27, *p ≤ 0.001, **p ≤ 0.0001. Cyt, cytosolic; Mit, mitochondrial; ANT, adenine nucleotide transporter; Dox, doxycycline.

*P75*^*NTR *^expression in tumor cells induces a dose-dependent increase of the pro-apoptotic members (Bad, Bax and Bak) and a decrease of the anti-apoptotic members (Bcl-2 and Bcl-XL) of the Bcl-2 family [47]. Since we were unable to directly measure the relative amounts of these proteins in mesDA neurons themselves, we followed up on this possibility by application of HA14-1 and chelerythrine chloride, inhibitors of Bcl-2 and Bcl-XL, respectively. HA14-1 was discovered as an inhibitor of Bcl-2 using a computer screening strategy based on its predicted structure [24]. Chelerythrine chloride was identified by a high-throughput screening of natural compounds [48]. Both are highly specific for their targets. If the levels of the two anti-apoptotic members of the Bcl-2 family of proteins are lowered, as suggested by these previous tumor cell-experiments [47], the sensitivity to HA14-1 and chelerythrine chloride should be higher when the level of *Engrailed *protein decreases. Twenty-four hours after treatment with HA14-1 and chelerythrine chloride, the rate of survival of mesDA neurons, derived from E12 *En2-/- *embryos, was, on average, 75.3 ± 6.8% and 92.7 ± 2.7% (*p *< 0.001, n = 7 for both compounds) higher, respectively, than their counterparts derived from *En*^*HT *^littermates (Figure 6A,B), suggesting that reduced *Engrailed *and elevated *P75*^*NTR *^expression lowers the threshold at which the intrinsic pathway of apoptosis is triggered.

**Figure 7:**
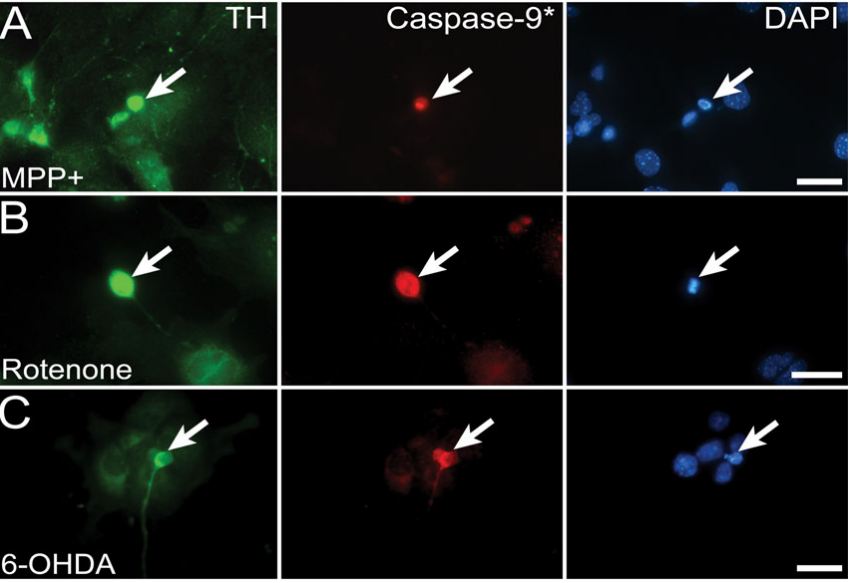
**Mitochondrial apoptosis after complex I inhibition**. **(A-C) **Immunohistochemistry of E12 ventral midbrain cell cultures. Caspase-9 is activated in wild-type mesDA neurons (arrows), treated with MPP+, rotenone, or 6-OHDA.

These results could indicate that the reduction in *Engrailed *expression increases the vulnerability of mesDA neurons to mitochondrial dysfunction in a general manner, since the three most commonly used reagents to model PD – MPP^+^, 6-Hydroxydopamine (6-OHDA) and rotenone [17,29,49,50] – also cause cell death by induction of this pathway of apoptosis, evident from activation of caspase-9 [51-53] (Figure 7A–C). To test this hypothesis directly, we treated *En*^*HT *^mesDA neurons with MPP^+^, the metabolite of MPTP, an inhibitor of complex-I of the mitochondrial electron transport chain [49]. After 48 hours in culture, the rate of survival of *En2-/- *mesDA neurons was, on average, 124.5 ± 5.0% (*p *< 0.001, n = 12) higher than for their *En1 *heterozygote (*En*^*HT*^) littermates, suggesting that MPTP and *Engrailed *may act upon the same molecular pathway upstream of caspase-9 (Figure 6A,B).

To determine whether this sensitivity to cell death is specifically related to instability of the mitochondria, we also employed Apoptosis Activator-2 [19], Prima-1 [27], and TNFα [31]. The first induces cell death by triggering apoptosome formation, the second by activation of p53 and the third, as a ligand of the TNF receptors, induces the extrinsic, receptor-mediated pathway of apoptosis. In all three cases, the level of *En1 *expression did not have a significant influence on the survival rates of these neurons (-8.1 ± 6.0% (*p *= 0.67) for Apoptosis Activator-2, 4.6 ± 6.2% (*p *= 0.82) for Prima-1, and 5.1 ± 3.1% (*p *= 0.48) for TNFα; n = 7 for all three experiments; Figure 6A,B).

### Gain of function in a mesDA cell line

To confirm the *Engrailed *dose-dependent sensitivity of mesDA neurons to mitochondrial insult, we performed a gain-of-function experiment using the inducible Tet-On system to express *En1 *in the dopaminergic cell line MN9D [54]. As in the primary cultures, *En1 *was protective against cell death if induced by administration of MPP^+ ^(42.4 ± 0.7%, *p *< 0.0001, n = 23), chelerythrine chloride (46.7 ± 1.3%, *p *< 0.0001, n = 10) and HA14-1 (32.4 ± 1.3%, *p *< 0.0001, n = 10). Accordingly, survival rate was not significantly altered if the other three reagents were employed (-10.7 ± 4.7% (*p *= 0.26) for Apoptosis Activator-2, -8.3 ± 5.2% (*p *= 0.50) for Prima-1, and 1.0 ± 7.4% (*p *= 0.95) for TNFα; n = 12 for all experiments; Figure 6C,D).

Functional inhibition of Bcl-2 and Bcl-XL and application of MPP^+ ^induces apoptosis by release of cytochrome C from the mitochondrial inter-membrane space into the cytosol [55,56]. The protective effect of *En1 *against HA14-1, chelerythrine chloride and MPP^+ ^may be attributable to higher mitochondrial stability. To test this hypothesis, we compared the cytosolic and mitochondrial protein fractions of *En1*-expressing MN9D cells to non-expressing cells. The proportion of cytochrome C in the mitochondria was always significantly higher after induction of *En1*, either in the presence of MPP^+ ^or in untreated control cultures (76.1 ± 7.3%, *p *= 0.03, n = 3, and 41.6 ± 7.0%, *p *= 0.005, n = 3, respectively), whereas the total amount of cellular cytochrome C was unaltered (Figure 6F,H). In contrast, the proportional levels in mitochondria and cytosol of the apoptosis inducing factor, which causes caspase-independent mitochondrial apoptosis [57], were independent of the level of *En1 *expression, suggesting that *Engrailed *participates in the regulation of mitochondrial stability via the cytochrome C/caspase-dependent pathway of apoptosis rather than the caspase-independent pathway, represented by apoptosis inducing factor.

## Discussion

In this study, we provide evidence that cell death in *Engrailed*-deficient mesDA neurons is a result of higher *P75*^*NTR *^expression and the loss of Erk1/2 activity. Furthermore, we show that the dose of *Engrailed *is part of the molecular mechanism that determines the sensitivity of these neurons to mitochondrial insult.

P75^NTR ^can cause apoptosis in various neuronal populations by mere high expression [10] or as a mediator of a (pro-)neurotrophin death signal [8]. Alternatively, since TrkB and C are expressed by the mesDA neurons, the abnormal increase in the expression of P75^NTR^ could introduce a neurotrophin dependency, which does not occur in the wild type at this age. The latter is more likely in mesDA neurons deprived of the *Engrailed *genes, since the death signal could be counteracted by addition of neurotrophins (BDNF, NT3, NT4) [58]. Furthermore, the Kd calculated from the dose response of BDNF, corresponds to the known affinity of neurotrophins for P75^NTR ^and Trk receptors [40], demonstrating that the survival effect of the neurotrophins is attributable to direct binding to the receptors on the surface of mesDA neurons, as opposed to a survival signal that originates from the surrounding cells, which would be reflected in a different shape of the dose response curve. A direct interaction of the neurotrophins with receptors on mesDA neurons is also consistent with our previous findings that the *Engrailed *genes are cell-autonomously required for the survival of these cells [11]. The ineffectiveness of Ngf can be readily explained by the lack of expression of TrkA. It is noteworthy that neither neurotrophins nor inhibition of P75^NTR ^completely (that is, 100%) rescue mesDA neurons deprived of *Engrailed *genes, and it is thus possible that P75^NTR ^is only one of many factors contributing to the death of mesDA neurons in the absence of the *Engrailed *genes. However, since the *in vitro *timeframe of loss of *En*^*DM *^mesDA neurons is 72 hours, it is also possible that those cells, which died anyway, had already been committed to cell death before the knock-down of *P75*^*NTR *^by the RNA duplexes was sufficiently high or the neurotrophin effect set in.

Neurotrophin-induced Trk/P75^NTR ^interaction, or lack thereof, can provoke intracellular activation of the MAPK and PI3K pathways. Among MAPKs, JNK and p38 participate in stress responses and often trigger apoptosis, while Erk1/2 signaling regulates cell proliferation, differentiation and survival [59]. Alternatively, the pro-survival role of neurotrophins can be mediated by PI3K signaling. This is in contrast to our findings. The activity of JNK, p38 or PI3K is independent of the *Engrailed *genes and the level of *P75*^*NTR *^expression in mesDA neurons. The lack of Erk1/2 activity in *En*^*DM *^mesDA neurons, which is reversed under any of the rescue conditions, demonstrates that the level of Erk1/2 phosphorylation in mesDA neurons is correlated with *Engrailed *expression and inversely to the level of P75^NTR^, suggesting that the P75^NTR ^death signal is mediated in these neurons by suppression of Erk1/2 activity. Although the association of P75^NTR ^and the Erk proteins has been shown in PC12 cells [60], regulation of the activity of the Erk1/2 signaling pathway as a result of death signaling by P75^NTR ^is a novel mechanism, which signifies both the role of sustained activity of Erk1/2 in the survival and/or of P75^NTR ^in the demise of mesDA neurons. Our data also suggest that the survival effect of neurotrophins may be the result of dis-inhibition of a death signal (high expression of *P75*^*NTR*^) that is triggered in the absence of the proper transcriptional regulation (by the *Engrailed *genes) during the course of development or possibly throughout life.

The pro-apoptotic role of P75^NTR ^has been established in the animal models of neurodegenerative disorders, other than PD, including beta-amyloid peptide-dependent cell death [61], or stress conditions, including ischemia [62]. Although function of the ligands and the co-receptors of P75^NTR^, TrkA/B/C, have been investigated in mesDA neurons [63-65], the role of P75^NTR^, itself, has not been reported in this system. Our work provides the first evidence for the pivotal role of *P75*^*NTR *^in mesDA neurons, as a negative mediating factor for the lifelong survival function of the *Engrailed *genes in this neuronal population.

The sensitivity to neurotoxin-induced cell death by MPTP and to inducers of mitochondrial instability, such as the Bcl-2 and Bcl-X_L _inhibitors, is inversely correlated with the dose of *Engrailed *expression in mesDA neurons. Mitochondrial dysfunction was first implicated in the pathogenesis of PD after accidental administration of MPTP by drug abusers and a consequent Parkinsonian syndrome. Lowered complex I activity and reduced mitochondrial stability [3] are believed to be key factors in the etiology of PD. In postmortem brains of PD patients, reduced activity of complex-I (NADH/ubiquinone oxidoreductase) of the mitochondrial electron transport chain [49] and elevated levels of the pro-apoptotic members of the Bcl-2 family have been observed in the substantia nigra [66]. Then, three genes, *DJ1*, *PINK1 *and *OMI*/*HTRA2*, mutations of which have been associated with familiar forms of PD, were discovered to have a function in the mitochondria [5]. Furthermore, the toxicity of MPTP is conferred by inhibition of complex I of the electron transport chain, triggering the release of cytochrome-C from the mitochondrial intermembrane space into the cytosol, which involves Bcl-2 family members [7]. Here, we demonstrate that the sensitivity to neurotoxin-induced cell death and to inducers of mitochondrial apoptosis, such as the Bcl-2 and Bcl-X_L _inhibitors, is inversely correlated to the dose of *Engrailed *expression in mesDA neurons. This, the previous findings of other groups, and the PD-like slow progressive loss of nigral dopaminergic neurons in *En*^*HT *^mice [14] suggest that the mode of action of the *Engrailed *genes converges with MPTP toxicity and possibly also with the disease mechanism on the mitochondria. Intriguingly, a recent association study into PD indicated a single nucleotide polymorphism in the intron of *En1 *as a potential risk factor for sporadic forms of this disease [67].

## Abbreviations

BDNF: brain-derived neurotrophic factor; E: embryonic day; En: Engrailed; *En*^*DM*^: *Engrailed *double mutant mice (*En1-/-;En2-/-*); *En*^*HT*^: heterozygous null for *En1 *and homozygous null for *En2 *(*En1+/-;En2-/-*); Erk: extracellular-signal-regulated kinase; GDF: growth differentiation factor; GDNF: glial cell line-derived neurotrophic factor; JNK: c-Jun N-terminal kinase; MAPK: mitogen-activated protein kinase; mesDA: mesencephalic dopaminergic; MPTP: 1-methyl-4-phenyl-,1,3,6-tetrahydropyridine; NGF: nerve growth factor; NT: neurotrophin; PD: Parkinson's disease; PI3K: phosphotidyl inositol-3 kinase; RNAi: RNA interference; siRNA: small interfering RNA; TGF: transforming growth factor; Trk: tropomyosin-receptor-kinase.

## Competing interests

The authors declare that they have no competing interests.

## Authors' contributions

KNA and HHS conceived of, designed and discussed the studies, carried out the experiments, analyzed and interpreted the data and wrote the draft and the final version of the manuscript. PS and LA made the stable MN9D cell lines. SS helped with immunoassays. The manuscript was approved by all authors.

## Supplementary Material

Additional file 1 **List of compounds and concentrations.** Compounds employed for the cell culture experiments: Final concentrations, solvents, duration of application, activity, source and literature. Click here for file
