# Supplementary material for: Elevated P75NTR expression causes death of engrailed-deficient midbrain dopaminergic neurons by Erk1/2 suppression
Source: Neural Dev. 2009 Mar 16;4:11. doi: 10.1186/1749-8104-4-11 (PMC2667502; doi:10.1186/1749-8104-4-11)
Supplement: Additional file 1 — List of compounds and concentrations. Compounds employed for the cell culture experiments: Final concentrations, solvents, duration of application, activity, source and literature. [file 1749-8104-4-11-S1.pdf]

List of compounds and concentrations

	Compound	Solvent	Final Conc.	Time	Activity	Reference	Source
1	6-OHDA	Medium	200µM	24h	mesDA specific neurotoxin	(Fink et al., 1992)	Sigma-Aldrich
2	Ac-DEVD-CMK	DMSO	1µM	72h	Caspase-3 inhibitor	(Mocanu et al., 2000)	EMD Biosciences
3	Apoptosis Activator 2	DMSO	200nM	24h	Apaf-1 oligomerization	(Nguyen and Wells, 2003)	EMD Biosciences
4	Bax-Inhibiting Peptide, V5	H_2O	100µM	72h	Ku70 Bax inhibiting	(Sawada et al., 2003	EMD Biosciences
5	Caspase Inhibitor II	DMSO	2.7µM	72h	Pan-Caspase inhibitor	(Thornberry and Lazebnik, 1998)	EMD Biosciences
6	Caspase-9 Inhibitor (Aldehyde)	DMSO	20µM	72h	Caspase-9 inhibitor	(Fadeel et al., 1999)	AG. Scientific
7	Chelarythrine Chloride	DMSO	4µM	18h	Bcl-Xl inhibiting factor	(Tanaka et al., 2004)	Alexis Biochemicals
8	HA14	DMSO, PBS	30µM	18h	Bcl2 inhibiting peptide	(Wang et al., 2000)	Alexis Biochemicals
9	MPP+	H_2O	33µM	24h	mesDA specific neurotoxin	(Langston et al., 1984)	Sigma-Aldrich
10	Pifithrin-α	DMSO	10µM	72h	p53 inhibitor	(Hoagland et al., 2005)	Sigma-Aldrich
11	Prima-1	H_2O	70µM	18h	p53 activator	(Bykov et al., 2002),	Alexis Biochemicals
12	Rex	PBS	20mg/ml	72h	P75 inhibitor	(Kruttgen et al., 1998)	Louis Reichardt
13	Rotenone	DMSO	40nM	24h	mesDA specific neurotoxin	(Testa et al. 2005)	Sigma-Aldrich
14	SP600125	DMSO	7µM	72h	JNK inhibitor	(Wang et al., 2004)	EMD Biosciences
15	TNF-α	PBS	5ng/ml	24h	Apoptosis inducer	(Adams, 2003)	Sigma-Aldrich
16	U0126	DMSO	400nM	72h	Mek inhibitor	(Duncia et al., 1998),	EMD Biosciences
17	Z-IE(OMe)TD(OMe)-FMK	DMSO	1µM	72h	Caspase-8 inhibitor	(Martin et al., 1998)	EMD Biosciences

Literature

Adams, J.M. (2003) Ways of dying/ multiple pathways to apoptosis. Genes Dev. *17*, 2481-95.

Bykov, V.J., N. Issaeva, A. Shilov, M. Hultcrantz, E. Pugacheva, P. Chumakov, J. Bergman, K.G. Wiman, and G. Selivanova. (2002) Restoration of the tumor suppressor function to mutant p53 by a low-molecular-weight compound. Nature medicine. *8*, 282-8.

Duncia, J.V., J.B. Santella, 3rd, C.A. Higley, W.J. Pitts, J. Wityak, W.E. Frietze, F.W. Rankin, J.H. Sun, R.A. Earl, A.C. Tabaka, C.A. Teleha, K.F. Blom, M.F. Favata, E.J. Manos, A.J. Daulerio, D.A. Stradley, K. Horiuchi, R.A. Copeland, P.A. Scherle, J.M. Trzaskos, R.L. Magolda, G.L. Trainor, R.R. Wexler, F.W. Hobbs, and R.E. Olson. (1998) MEK inhibitors: the chemistry and biological activity of U0126, its analogs, and cyclization products. Bioorg. Med. Chem. Lett. *8*, 2839-44.

Fadeel, B., Z. Hassan, E. Hellstrom-Lindberg, J.I. Henter, S. Orrenius, and B. Zhivotovsky. (1999) Cleavage of Bcl-2 is an early event in chemotherapy-induced apoptosis of human myeloid leukemia cells. Leukemia. *13*, 719-28.

Fink, D.W., P.T. Horn, and B.L. Mirkin. (1992) Differential effects of 6-hydroxydopamine-induced ablation of peripheral and central adrenergic axons on C-1300 murine neuroblastoma tumor growth and catecholamine concentration in the A/J mouse. The Journal of pharmacology and experimental therapeutics. *262*, 1070-5.

Hoagland, M.S., E.M. Hoagland, and H.I. Swanson. (2005) The p53 inhibitor pifithrin-alpha is a potent agonist of the aryl hydrocarbon receptor. The Journal of pharmacology and experimental therapeutics. *314*, 603-10.

Kruttgen, A., J.C. Moller, J.V. Heymach, Jr., and E.M. Shooter. (1998) Neurotrophins induce release of neurotrophins by the regulated secretory pathway. Proc. Natl. Acad. Sci. U S A. *95*, 9614-9.

Langston, J.W., I. Irwin, E.B. Langston, and L.S. Forno. (1984) 1-Methyl-4-phenylpyridinium ion (MPP+): identification of a metabolite of MPTP, a toxin selective to the substantia nigra. Neuroscience letters. *48*, 87-92.

Martin, D.A., R.M. Siegel, L. Zheng, and M.J. Lenardo. (1998) Membrane oligomerization and cleavage activates the caspase-8 (FLICE/MACHalpha1) death signal. The Journal of biological chemistry. *273*, 4345-9.

Mocanu, M.M., G.F. Baxter, and D.M. Yellon. (2000) Caspase inhibition and limitation of myocardial infarct size: protection against lethal reperfusion injury. British journal of pharmacology. *130*, 197-200.

Nguyen, J.T., and J.A. Wells. (2003) Direct activation of the apoptosis machinery as a mechanism to target cancer cells. Proc. Natl. Acad. Sci. U S A. *100*, 7533-8.

Sawada, M., W. Sun, P. Hayes, K. Leskov, D.A. Boothman, and S. Matsuyama. (2003) Ku70 suppresses the apoptotic translocation of Bax to mitochondria. Nat. Cell Biol. I, 320-9.

Tanaka, K., M. Honda, and T. Takabatake. (2004) Anti-apoptotic effect of atorvastatin, a 3-hydroxy-3-methylglutaryl coenzyme a reductase inhibitor, on cardiac myocytes through protein kinase C activation. Clin. Exp. Pharmacol. Physiol. *31*, 360-4.

Testa, C.M., T.B. Sherer, and J.T. Greenamyre. (2005) Rotenone induces oxidative stress and dopaminergic neuron damage in organotypic substantia nigra cultures. Brain Res. Mol. Brain Res. *134*, 109-18.

Thornberry, N.A., and Y. Lazebnik. (1998) Caspases: enemies within. Science. *281*, 1312-6.

Wang, J.L., D. Liu, Z.J. Zhang, S. Shan, X. Han, S.M. Srinivasula, C.M. Croce, E.S. Alnemri, and Z. Huang. (2000) Structure-based discovery of an organic compound that binds Bcl-2 protein and induces apoptosis of tumor cells. Proc. Natl. Acad. Sci. U S A. *97*, 7124-9.

Wang, W., L. Shi, Y. Xie, C. Ma, W. Li, X. Su, S. Huang, R. Chen, Z. Zhu, Z. Mao, Y. Han, and M. Li. (2004) SP600125, a new JNK inhibitor, protects dopaminergic neurons in the MPTP model of Parkinson's disease. Neurosci. Res. *48*, 195-202.
